# Different models and single-nucleotide polymorphisms signal the simulated weak gene-gene interaction for a quantitative trait using haplotype-based and mixed models testing

**DOI:** 10.1186/1753-6561-3-s7-s77

**Published:** 2009-12-15

**Authors:** Ilija P Kovac, Marie-Pierre Dubé

**Affiliations:** 1 Research Centre of the Montreal Heart Institute, 5000 Belanger - C1443, Montreal (Quebec) H1T 1C8, Canada; 2 Université de Montréal, Faculté de Médecine, Pavillon Roger-Gaudry, 2900, Boulevard Edouard-Montpetit, Montreal (Quebec) H3T 1J4, Canada

## Abstract

Knowledge of simulated genetic effects facilitates interpretation of methodological studies. Genetic interactions for common disorders are likely numerous and weak. Using the 200 replicates of the Genetic Analysis Workshop 16 (GAW16) Problem 3 simulated data, we compared the statistical power to detect weak gene-gene interactions using a haplotype-based test in the UNPHASED software with genotypic mixed model (GMM) and additive mixed model (AMM) mixed linear regression model in SAS. We assumed a candidate-gene approach where a single-nucleotide polymorphism (SNP) in one gene is fixed and multiple SNPs are at the second gene. We analyzed the quantitative low-density lipoprotein trait (heritability 0.7%), modulated by simulated interaction of rs4648068 from 4q24 and another gene on 8p22, where we analyzed seven SNPs. We generally observed low power calculated per SNP (≤ 37% at the 0.05 level), with the haplotype-based test being inferior. Over all tests, the haplotype-based test performed within chance, while GMM and AMM had low power (~10%). The haplotype-based and mixed models detected signals at different SNPs. The haplotype-based test detected a signal in 50 unique replicates; GMM and AMM featured both shared and distinct SNPs and replicates (65 replicates shared, 41 GMM, 27 AMM). Overall, the statistical signal for the weak gene-gene interaction appears sensitive to the sample structure of the replicates. We conclude that using more than one statistical approach may increase power to detect such signals in studies with limited number of loci such as replications. There were no results significant at the conservative 10^-7 ^genome-wide level.

## Background

With efforts to uncover more genetic variation for common polygenic disorders, there is accrued interest in the analysis of genetic interactions [1]. It is very plausible to expect a plurality of statistical genetic interactions with each having a small effect, rather than a few strong interactions. Here, we compare the statistical power of several methods to detect a weak gene-gene interaction for a quantitative trait in a combined data set that includes both familial and unrelated samples, in a hypothetical candidate-gene design in which the single-nucleotide polymorphism (SNP) in one gene is fixed (for example, as replicated by marginal association analysis and/or for a known functional factor) and multiple SNPs are at the second interacting candidate gene. We used the Genetic Workshop Analysis 16 (GAW16) Problem 3 simulated data set [2] with knowledge of the modeling scheme. Using 200 replicates, we compared the power of the haplotype-based test in the computer program UNPHASED [3] to that of genotypic and additive mixed models (GMM and AMM) as implemented with a mixed linear regression model in SAS.

## Methods

### Subjects and phenotype

The subjects consist of 6,476 related and unrelated individuals from the Framingham Heart Study as made available for the GAW16 Problem 3 simulated data by dbGAP. We used two phenotype definitions in separate analyses. A first quantitative phenotype that was used for haplotype-based analyses only was the covariate-adjusted multivariate linear regression residual of the low-density lipoprotein (LDL) variable, averaged over the three visits and standardized. The covariates used for the adjustment were sex, age, diet, medication use, high-density lipoprotein (HDL), triglyceride level, and smoking. The main quantitative phenotype used for both haplotype-based and mixed model analyses was the LDL measurement at the first visit, regression-adjusted for age and sex only (excluding subjects on lipid-lowering medication). The simulated LDL measurement at the first visit did not show notable departure from the normal distribution in the first replica, suggesting that non-normality would not be an important factor in this study.

### SNP data

**Figure 1:**
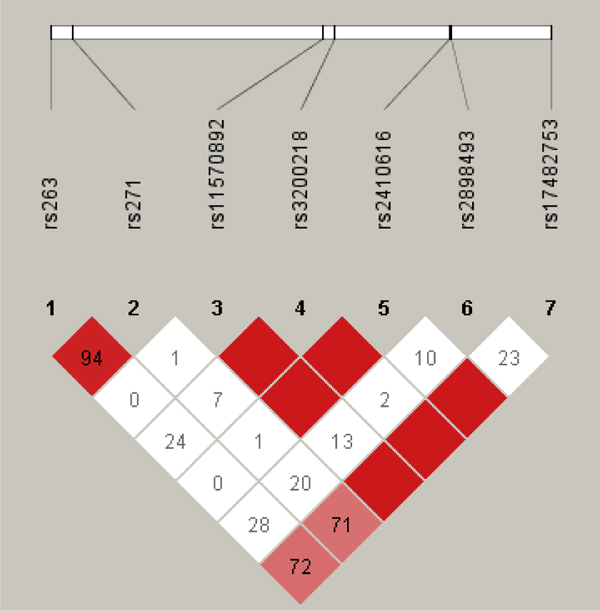
**Haplotype structure in the *LPL *gene region at 8p22**.

The SNP rs4648068 in the *NFKB1 *(nuclear factor kappa-B, subunit 1) gene at chromosome 4q24 was fixed in the analyses: it is involved in the simulated gene-gene interaction with the lipoprotein lipase precursor (*LPL*) gene with 0.7% heritability for the *LDL *trait. Seven SNPs from the 500 k chip, with minor allele frequencies ranging from 0.07-0.22, were selected in the *LPL *gene region at 8p22 as per HapMap gene position 19,841,058 to 19,869,049, encompassing an additional 10 kb on each side as available. These SNPs are: rs263, rs271, rs11570892, rs3200218, rs2410616, rs2898493, and rs17482753; respective chromosomal positions are 19856900, 19857092, 19857982, 19867897, 19868351, 19872959, 19873001, and 19876926. Figure 1 presents haplotype structure in this ~20-kb region based on the *D*' measure of linkage disequilibrium obtained using 849 unrelated subjects in the computer program Haploview [4].

### Statistical analysis

#### Haplotype-based analysis with UNPHASED

The UNPHASED software implements a likelihood approach for primarily haplotype-based analysis of data, which can include both familial and unrelated subjects [3]. The test for gene-gene interaction for a quantitative trait that we used compares the null hypothesis (H_0_) of equal contributions for all gene combinations (in haplotype form) sharing the same alleles at the conditioning marker, versus the alternative hypothesis (H_1_) of differential multiplicative contributions from the test marker. The test uses a likelihood-ratio chi-square statistics to compare models with and without the interaction terms. The frequency of gene combinations was limited to 0.01. Gene-gene interaction using SNP-SNP testing (in haplotype form) was evaluated between rs4648068 and each SNP in the *LPL *gene region one at a time. Gene-gene interaction was also evaluated using SNP-haplotype testing between rs4648068 and each consecutive pair of SNPs in the *LPL *gene region one pair at a time as haplotypes, e.g., rs263-rs271, rs271-rs11570892, etc. The SNP-haplotype testing was conducted only in the analysis of the first phenotype.

#### Mixed model analysis with SAS

We used the mixed linear regression model implemented in PROC MIXED in SAS version 9.1.3 (SAS Institute Inc., Cary, NC, USA). To evaluate significance of the genotypic SNP-SNP interaction in the presence of potential marginal genotypic effects, we used the following full model for the main LDL phenotype and each of seven SNPs from the *LPL *gene region:

where LDL is measured at the first visit excluding subjects on lipid medication, SNP_0 _is the fixed rs4648068, SNP_*i *_is from the *LPL *gene region (*i *= 1-7), and pedno is the pedigree ID included as a random effect to control for familial dependence. For the GMM test, SNPs were represented by three genotype classes (two homozygotes and a heterozygote). For the AMM test, the number of minor alleles in a genotype was coded as 0, 1, or 2. We used Type 3 tests of fixed effects to evaluate statistical significance.

## Results

### Haplotype-based SNP-SNP versus SNP-haplotype testing

In our first haplotype-based analyses of 200 replicates with the first LDL definition, there was low power (≤ 15%) at the 0.05 significance level with markers rs3200218 and rs2898493 in either SNP-SNP tests or SNP-haplotype tests involving these markers (not shown). The SNP-SNP testing was more powerful than SNP-haplotype testing (maximal power 15% at rs3200218 vs. 9.5% at haplotype rs11570892-rs3200218).

### Haplotype-based SNP-SNP testing versus genotypic and additive mixed model testing

Table 1 presents power estimates for the main LDL phenotype definition using SNP-SNP interaction testing with UNPHASED and the mixed models with SAS. With UNPHASED, there was slightly higher power at rs3200218 as compared with the first phenotype definition noted above (17% vs. 15%). While generally low, particularly so at the 0.01 level, the power to detect the interaction calculated per SNP was greater with the mixed models (maximum of 37% vs. 17% at the 0.05 level). Each of the mixed models produced interaction signals at SNPs at opposite ends of the *LPL *gene region, while UNPHASED produced an interaction signal only with rs3200218 located between these SNPs, which is the only SNP to have a detectable marginal effect in either GMM or AMM without the interaction signal. The GMM and AMM produced an opposite pattern of interaction signals at rs263 and rs271, and both produced a signal at rs17482753. The AMM detected marginal SNP effects more frequently than GMM. When calculated over all SNP tests, the haplotype-based test had no power to detect the interaction, while both GMM and AMM had low power.

**Table 1:** Statistical power (%) for haplotype-based and mixed model analyses of the Covariate-adjusted LDL measurement in 200 replicates

			Mixed model^b^							
			
	Haplotype-based^a^		rs4648068 × SNP				Marginal effect			
			
	rs4648068 × SNP^c^		Genotype analysis		Additive genotype recoding		Genotype analysis		Additive genotype recoding	
					
SNP	*α *= 5%	1%	5%	1%	5%	1%	5%	1%	5%	1%
rs4648068	-	-			-	-	**13^e^**	**2.57**	**44.5**	**16.9**
rs263	1	0	2.5	0	**21.5**	**4.5**	**11**	1	**88**	**58.5**
rs271	3	0	**37**	**15.5**	2.5	0	**21**	**5**	**45.5**	**18**
rs11570892	0.5	0	2	0	1.5	0	**17**	**2**	**60**	**27**
rs3200218	**17**	**3.5**	5	0	3.5	0	**95.5**	**80.5**	**86.5**	**57**
rs2410616	0.5	0	0.5	0	3	0	**14**	**3**	**72**	**37.5**
rs2898493	4.5	1	9	1.5	1	0	3.5	0	0.5	0
rs17482753	1	0	**20**	**5**	**32.5**	**7**	2	0	**63**	**25.5**
Overall^d^	3.9	0.6	**10.9**	**3.14**	**9.36**	**1.64**	**18.2**	**7.82**	**51.9**	**24.4**

^a^Haplotype-based analyses with UNPHASED

^b^Mixed model SAS

^c^Interaction term

^d^Proportion of significant *p*-values from 1400 tests (7 SNPs tested per each of 200 replicates)

^e^Bold font indicates greater than expected for the corresponding significance level.

### Signal identification by replicates with different models

**Figure 2:**
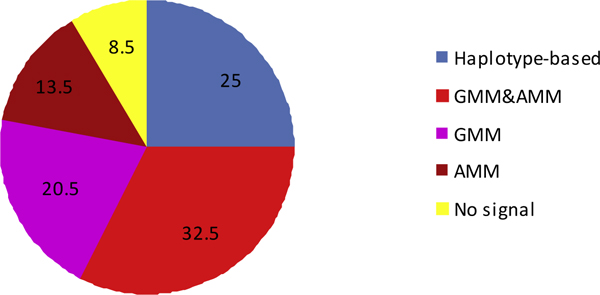
**Percentage of replicates with any *p *< 0.05 SNP-SNP interaction (*n *= 200 replicates)**. GMM, genotypic mixed model; AMM, additive mixed model.

Figure 2 shows the percent of replicates, calculated out of 200 replicates, which show a significant gene-gene interaction result at the 0.05 level with at least one of the tested SNPs, for the different models. The haplotype-based test produced a signal in a unique set of replicates, while the GMM and AMM featured some shared and some method-specific replicates. Taken together, these methods identified the gene-gene interaction in 91.5% of the replicates.

## Discussion

We generally observed low statistical power to detect a weak simulated candidate gene-gene interaction for the LDL trait with heritability of 0.7%, where one SNP was fixed and multiple SNPs are at the second gene. There was a small increase in power per SNP with the haplotype-based tests when LDL was not adjusted for HDL and triglyceride level, potentially retaining useful variation (the LPL candidate gene is simulated to contribute to HDL with a heritability of 0.003). In further analyses the haplotype-based test was inferior to GMM or AMM, particularly when power was calculated over all SNP tests.

The haplotype-based test detected gene-gene interaction with a single SNP in the region, while GMM and AMM had shared and specific SNPs. We also examined the relationship between the specific methods used and the replicates that produced any gene-gene interaction signals. While there was some overlap in successful replicates with the GMM and AMM models, each of the three models identified distinct replicates, for a total of 183/200 replicates producing a detectable gene-gene interaction signal. Notably, the haplotype and mixed model tests did not share a single successful replica. The sole SNP identified with low power in the haplotype-based interaction test was also the only one with a robust marginal effect in both the GMM and AMM (without the interaction signal). Accordingly, the interaction signal from this particular SNP could be obscured in the mixed models by the strong marginal effect. The inability of the haplotype-based test to detect the weak interaction at other SNPs compared with mixed models suggests overall inferiority of the former approach for this simulated genetic interaction. In this data set, the weak gene-gene interaction followed different genetic patterns in different replicates, and its detection was sensitive to the particular structure of the replicates.

## Conclusion

The systematic use of more than one analytical approach may help to increase power to identify a weak gene-gene interaction in a limited number of test loci such as in a replication study. The replication sample may not necessarily present an identical genetic pattern to that of the original study. It might be expected that sensitivity to sample structure would be less of an issue for stronger gene-gene interactions. Factors beyond statistical interaction patterns, such as the presence of marginal genetic effects, may decrease the power to detect genetic interaction in mixed models. Finally, we note that not a single result from any analysis was significant at the conservative 10^-7 ^genome-wide level for a 500 k chip, indicating that these or similarly powered approaches would not detect such a weak gene-gene interaction from a genome-wide scan.

## List of abbreviations used

AMM: Additive mixed model; GAW16: Genetic Analysis Workshop 16; GMM: Genotypic mixed model; HDL: High-density lipoprotein; LDL: Low-density lipoprotein; LPL: Lipoprotein lipase precursor; SNP: Single-nucleotide polymorphism

## Competing interests

The authors declare that they have no competing interests.

## Authors' contributions

IPK performed statistical analysis, drafted the manuscript, and participated in the study design and interpretation. MPD participated in the study design, interpretation, and finalization of the manuscript.
